# Evidence-based practice guideline of Chinese herbal medicine for primary open-angle glaucoma (qingfeng -neizhang)

**DOI:** 10.1097/MD.0000000000010126

**Published:** 2018-03-30

**Authors:** Yingxin Yang, Qiu-yan Ma, Yue Yang, Yu-peng He, Chao-ting Ma, Qiang Li, Ming Jin, Wei Chen

**Affiliations:** a Department of Ophthalmology, Beijing Traditional Chinese Medicine Hospital, Capital Medical University; b Department of Ophthalmology, China-Japan Friendship Hospital; c Centre for Evidence-Based Chinese Medicine, Beijing University of Chinese Medicine, Beijing, China.

**Keywords:** Chinese herbal medicine, Chinese proprietary herbal medicine, clinical guideline, open-angle glaucoma, randomized controlled trials

## Abstract

**Background:**

Primary open angle glaucoma (POAG) is a chronic, progressive optic neuropathy. The aim was to develop an evidence-based clinical practice guideline of Chinese herbal medicine (CHM) for POAG with focus on Chinese medicine pattern differentiation and treatment as well as approved herbal proprietary medicine.

**Methods:**

The guideline development group involved in various pieces of expertise in contents and methods. Authors searched electronic databases include CNKI, VIP, Sino-Med, Wanfang data, PubMed, the Cochrane Library, EMBASE, as well as checked China State Food and Drug Administration (SFDA) from the inception of these databases to June 30, 2015. Systematic reviews and randomized controlled trials of Chinese herbal medicine treating adults with POAG were evaluated. Risk of bias tool in the Cochrane Handbook and evidence strength developed by the GRADE group were applied for the evaluation, and recommendations were based on the findings incorporating evidence strength. After several rounds of Expert consensus, the final guideline was endorsed by relevant professional committees.

**Results:**

CHM treatment principle and formulae based on pattern differentiation together with approved patent herbal medicines are the main treatments for POAG, and the diagnosis and treatment focusing on blood related patterns is the major domain.

**Conclusion:**

CHM therapy alone or combined with other conventional treatment reported in clinical studies together with Expert consensus were recommended for clinical practice.

## Abstract 2

Supplemental Digital Content is available in the text

## 1 Introduction

### 1.1 Objectives and scope of the guideline

Primary open-angle glaucoma (POAG) is a chronic, progressive optic neuropathy, and pathological high intraocular pressure is one of the important factors that results in optic nerve injury.^[1]^ At present, open-angle glaucoma is the world's first irreversible blinding disease. Incidence rate of open-angle glaucoma among people aged over 30, 40, and 50 years was 0.57%, 2.07%, and 0.29%, respectively.^[2,3]^ The prevalence of POAG increased with age. Obviously, the prevalence of open-angle glaucoma is very high. With the advance of the aging of China's population, the number of open-angle glaucoma patients will also increase.

Open-angle glaucoma (Qingfeng Neizhang) refers to a chronic eye disease, the main clinical manifestations of which are intermittent eye inflation, hypopsia, narrowness of visual field, and pale blue pupil.^[4]^ POAG is characterized by acquired optic nerve atrophy and loss of retinal ganglion cells and axon; the angle remains open when intraocular pressure is elevated. It was categorized as “Qingfeng Neizhang” in traditional Chinese medicine (TCM).

With the wide use of TCM for treating POAG in China, various clinical studies and reviews on this disease have been generated, which include expert opinion and experience, clinical practical reports, cohort study, randomized controlled trials (RCTs), and other types of studies. Some herbal medicines may have beneficial effects, but still there were some issues such as low quality and potential bias in those clinical trials.

This guideline was mainly formulated on the basis of evidence-based research on the treatment of POAG with TCM, combined with Expert consensus. The target patient population is those with POAG that visual field defects and optic nerve damage have already occurred but not yet progressed to optic atrophy. It is a reference for Chinese medicine ophthalmologist and other doctors from relevant departments. Its main purpose is to recommend evidence-based TCM treatment and medication for open-angle glaucoma, and to guide clinicians toward normative use of Chinese medicine in practice so as to provide a reference for clinical medication.

## 2 Methods

### 2.1 Methods used for establishing the guideline

#### 2.1.1 Literature inclusion criteria

(1) Research type: Systematic reviews and RCTs, regardless of whether blind method is used.
(2) Object of observation: Cases clearly diagnosed as open-angle glaucoma. Diagnostic criteria were proposed after the reference of early diagnostic criteria for primary glaucoma established by the national glaucoma collaboration group, 1987, which needs to meet the following conditions;
  ① Anterior chamber angle is open.
  ② Glaucomatous visual field changes.
  ③ Intraocular pressure >21 mm Hg (2.79 kPa).
  ④ Glaucomatous optic papilla damage and (or) retinal nerve fiber layer defects (confirmed by HRT/OCT examination).
  Among the above 4 items, 1, 2 and either one of 3, 4 are necessary.
(3) Interventions include the following methods: TCM injection, proprietary Chinese medicine, TCM decoction, acupuncture or integrated traditional Chinese and western medicine therapy.
(4) The controlled drugs included western medicine, placebo, or other medications for symptomatic treatment.
(5) The main outcome measures were intraocular pressure, visual field, and retinal nerve fiber layer thickness. Visual function was used as a criterion of secondary efficacy.

#### 2.1.2 Literature exclusion criteria

(1) Literature was the application of TCM combined with western medicine, and literatures that implicate inconsistent western medicine were used in the test scheme and the control scheme.
(2) If the author and the content of the same paper at the same time appear in conference papers and journal papers, excluding Conference Papers.
(3) If the author and the content of the same paper at the same time appeared in 2 or more than 2 papers, excluding the time after publication of the literature.

### 2.2 Method and process of retrieval

#### 2.2.1 Methods of literature search

Keywords are “primary open-angle glaucoma, simple glaucoma, glaucoma, cyanophthalmitis and glaucoma.” Meanwhile, TCM, herbal medicine, Chinese patent medicine, and randomized control are the free words, expression. After establishing retrieval expression, information can be searched from the electronic database. The specific retrieval expression is shown in annex 1. The start date is the date of database founded, and the deadline is June 30, 2015.

#### 2.2.2 Electronic database

The Chinese documentation database mainly includes CNKI, VIP, Chinese Biomedical Database (CBM), Wanfang Database, and TCM Ancient Books Database. The English documentation database mainly includes PubMed, Cochrane Library, Evidence-based medicine database, etc.

#### 2.2.3 Monographs

“Encyclopedia of ophthalmology of traditional Chinese medicine,” “Today's ophthalmology of traditional Chinese medicine,” “Ophthalmology of traditional Chinese medicine,” “Ophthalmology,” etc.^[4–7]^

#### 2.2.4 Criteria

“Chinese medicine diagnostic criteria of ophthalmic disorders,” “Chinese medicine diagnosis and treatment project for 95 diseases of 22 professions,” “Guidelines for diagnosis and treatment of common ophthalmic diseases in Traditional Chinese Medicine,” etc.^[8–10]^

#### 2.2.5 The retrieval results

**Figure 1:**
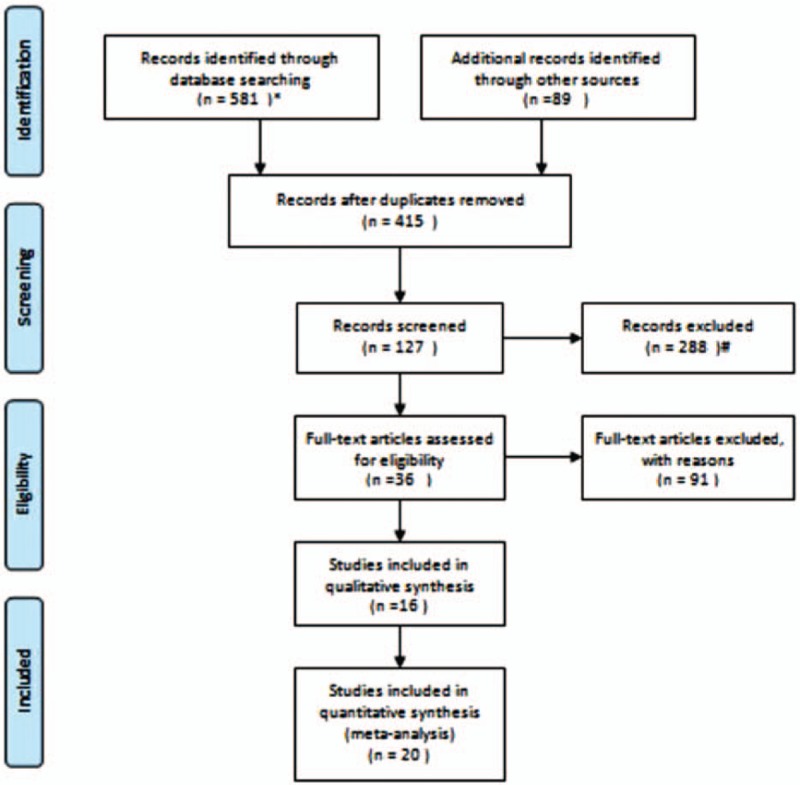
PRISMA 2009 flow Diagram. Note: ^∗^Records identified through database searching (n = 581): The Chinese literatures included 138 articles from Wanfang database, 91 from VIP database, 300 from CNKI, 52 from CBM. ^†^Records excluded (n = 288): Through reading the title and abstract, a total of 288 articles were excluded. There were 85 articles unrelated to interventions, 72 articles failed to conform to the articles, 41 articles were not complied with, and 90 articles were not studied.

By applying the Noteexpress software to screen the literature, this part of the work by 2 individuals was completed independently; after the completion of the verification, the views of the inconsistent literature are reviewed and judged by the third-party to remain or not. Finally, we found a total of 3 systematic reviews and found that 36 RCTs met the inclusion criteria.^[11–49]^ Details will be shown in PRISMA flow Diagram, Fig. 1.

#### 2.2.6 Evidence grading---GRADE standard

(1) High: We strongly believe that the real effect value approximates the effect estimate;
(2) Moderate: We have moderate confidence in the effect estimate; the real value may approximate the estimate, but there is still a possibility that they are not quite the same;
(3) Low: We have limited confidence in the effect estimates; the real value may differ significantly from the estimate;
(4) Very low: We almost have no confidence in the effect estimate; the real value is quite different from the estimate.
(5) Expert consensus: If it is a historical record of a therapy and clinical experience without clear modern literature support, it should be marked as Expert consensus, through expert discussion, behind the quality of evidence. In the process of Expert consensus, the opinions of the experts are divided into ① agree completely; ② agree, but have certain reservations; ③ agree, but have greater reservations; ④ disagree, but have reservations; ⑤ completely disagree. If the number of ① + ② accounted for more than two-third, or ① + ② + ③ >85%, then the Expert consensus is considered to be obtained, which would be adopted as a term.

#### 2.2.7 Rules for making GRADE recommendations grading criteria

(1) Highly recommended: Meet the following conditions at the same time: The advantages of the interventions obviously outweigh the disadvantages, evidence quality level is high or moderate, the medical cost is low, the intervention is easy to implement and operate, the drug side effect is small, and the risk is low.
(2) Weakly recommended: Meet the following conditions at the same time: The advantages of the interventions obviously outweigh the disadvantages, and also belong to one of the following circumstances, which is weakly recommended: High medical costs; The interventions are not available in all medical institutions; Historical records of therapy and clinical experience, which have obtained Expert consensus, without modern literature evidence support; Evidence quality level is low, but the Expert consensus is obtained. The above circumstances are weakly recommended.
(3) Strongly not recommended: The disadvantages of the interventions significantly outweigh the advantages, evidence quality level is high or moderate, and no Expert consensus is obtained.
(4) Weakly not recommended: Advantages and disadvantages of the interventions are not clear, evidence quality level is low or very low, and no Expert consensus is obtained.^[50]^

#### 2.2.8 The argumentation process of the guide

First of all, retrieval, evaluation, and summarization of literatures related to treatment of open angle glaucoma with TCM were carried out before meta-analysis was performed on literatures with high homogeneity. On the basis of the literature query and collation, a questionnaire survey on some professional issues that occurred during the guide development process was conducted according to evidence-based medicine. First, questionnaires and discussions were conducted by the open-angle glaucoma guideline development team experts to formulate group opinions, and then extensive expert questionnaire was conducted. The total number of experts in each questionnaire was not less than 30. (The list of experts is shown in annex 2.) Sum up the number of consents for experts, if it was greater than two-third, then the term was adopted and could be included in the guide. And 4 rounds of expert questionnaires were completed successively. After the questionnaire, the expert opinions were collected before a conference of expert argumentation was held, in which an agreement on common TCM syndrome differentiation and treatment medication was reached. And the first draft was formed in November 2015. Case verification was conducted during November to December 2015, and it was over by the end of December 2015. The experts were reorganized for a guideline symposium before the guidelines were further modified and improved according to case verification and expert opinions.

The revised draft guidelines were submitted to the ophthalmic specialist guidance group in May 2016 to be approved by the expert guidance group. In June 2016, 2 methodology experts and 2 clinical experts who were not in the guide group were invited to review the guidelines again and conduct an AGREE II evaluation. After the evaluation, the guidelines were submitted to the Ophthalmic Branch of China Association of Traditional Chinese Medicine to be reviewed, and after the approval, the final draft guidelines were formed.

## 3 Results

We found a total of 3 systematic reviews (literatures), but we found that the quality of these reviews was low and there was “such problems as the merging of highly heterogeneous studies.” Therefore, we did not directly cite the results. However, the references have been sorted out to offset for the lack of our literature search.

Referring to 36 RCTs, we used Revman5.3 software to conduct meta-analysis and evaluation of the risk of bias, and completed the quality evaluation of the evidence by using Gradepro software. See annex 3 table1. Characteristics of included RCTs and table2. Chinese herbal medicines tested in the included trials; See annex 4: Meta-analysis results; AS well as annex 5: Quality assessment of evidence.

### 3.1 Pattern classification criteria

#### 3.1.1 Syndrome of stagnation of liver Qi

Successive or simultaneous incidence in the both eyes, eyes swelling with headache, blurred vision, increased intraocular pressure, narrowed visual field, irritability or depression, chest tightness, vexation and irritability, red tongue with thin moss, and pulse string.

#### 3.1.2 Blood stasis and fluid retention syndrome

Unclear vision, headache eye swelling, elevated intraocular pressure, purple expansion of Baijing vein, dark purple tongue or tongue with ecchymosis and pulse string.

#### 3.1.3 Phlegm dampness syndrome

Increased intraocular pressure, dizziness and swelling, chest tightness nausea, poor appetite, pale or red tongue, greasy fur, pulse slip, or slip number.

#### 3.1.4 Syndrome of Yin deficiency of liver and kidney

The patient is usually ill for a long time with gradually scattered pupil, unclear vision, narrow visual object, swelled and hardened eyes, pale optic disc, and may be accompanied by insomnia and amnesia, soreness and weakness of waist and knees, red tongue with less fur, and thready rapid pulse.

### 3.2 Treatment protocol of Chinese herbal medicine (CHM)

#### 3.2.1 Treatment principles

In the early and middle stage (Humphrey mean defect [MD] value is larger than -12 dB) of the treatment, methods such as promoting Qi flow and soothing liver, invigorate the circulation of the water and blood, phlegm-and dampness-relieving were mainly used; And in the late stage (Humphrey MD value is less than or equal to -12 dB), the main methods were intermingled deficiency and excess syndrome, tonifying the liver and kidney and activating blood to promote diuresis were mainly used; Note that the disease can not only be manifested as blood stasis and fluid retention syndrome at some stages but also in the whole process of disease, and activating blood to promote diuresis medicine should be added in the treatment.

#### 3.2.2 Syndrome differentiation and treatment

##### 3.2.2.1 Syndrome of stagnation of liver Qi

Therapy: Promoting Qi flow and soothing liver

Main prescription: Dan Zhi Xiaoyaosan (”Internal Medicine Abstracts”) addition and subtraction.^[41]^ (Evidence level: very low, Recommendation level: Slightly recommended)

Commonly used drugs: bupleurum, angelica, white peony root, poria, atractylodes, licorice, paeonol, gardenia, prunella, salvia, and safflower.

Supporting evidence: RCTs have been conducted by Xie ^[41]^ where intraocular depressurization drops and vitamins were taken in controlled group, compared with Dan Zhi Xiaoyaosan and as experimental subjects group for 16 weeks. The result showed improvement in vision, intraocular pressure, and eyesight in latter group. Evidence level: Very low (see Meta-analysis results -comprision1, SOF table-1). Dan Zhi Xiaoyaosan has the tradition to deal with stagnation of liver Qi. Also, there is no adverse effect mentioned, and it is easy to take and low risk and cost. Recommendation level: Slightly recommended.

##### 3.2.2.2 Blood stasis and fluid retention syndrome (Expert consensus)

Therapy: Invigorate the circulation of the water and blood (Expert consensus)

Prescription: Taohong Siwu decoction (”YiZong Jin Jian”) and Wuling Powd (”Shanghan Lun”) addition and subtraction. (Expert consensus)

Commonly used drugs: peach kernel, safflower, shengdi, angelica, salvia, chuanxiong, red peony, plantago, alisma, poria, polyporus, atractylodes, guizhi, bupleurum

##### 3.2.2.3 Phlegm dampness syndrome

Therapy: Eliminate Damp and Sputum, Harmonizing stomach for descending adverse qi

Main prescription: Wendan Tang (”Beiji Qianjin Yaofang”) addition and subtraction. (Level of evidence: Expert consensus, Recommendation intensity: Weakly recommended)

Commonly used drugs: act pinellia, citrus, poria, licorice, citrus aurantium, bamboo ru, prunella vulgaris, fructus viticis.

##### 3.2.2.4 Syndrome of Yin deficiency of liver and kidney

Therapy: Tonifying the liver and kidney

Main prescription: Lycii and Chrysanthemi and Rehmanniae Bolus (“Yi Ji”) addition and subtraction.^[29,36]^ (Evidence level: very low, Recommendation level: Slightly recommended).

Commonly used drugs: rehmannia, cornus, yam, alisma, poria, paeonol, wolfberry, chrysanthemum, Salvia, turmeric.

Supporting evidence: RCTs have been conducted by Liu ^[36]^ where Lycii and Chrysanthemi and Rehmanniae Bolus was taken as experimental subjects compared with cobamamide in the controlled group. The result showed that improvements are significant in both groups. Evidence level: very low. (see Meta-analysis results -comparison 2 and SOF table-2). RCTs have been conducted by Huang^[29]^ where Timolol eye drops were taken in controlled group compared with Lycii and Chrysanthemi and Rehmanniae Bolus as experimental subjects group. The result showed better improvements in MD, mean sensitivity (MS), and intraocular pressure in the latter group. Evidence level: very low. (see Meta-analysis results - comparison 3, and SOF table-3).

Although both evidence levels are very low, being traditional medicine for Yin deficiency of liver and kidney, Lycii and Chrysanthemi and Rehmanniae Bolus is low cost and easy to find without any adverse effect. Recommendation level: Slightly recommended.

##### 3.2.2.5 Proprietary Chinese medicine

Ginkgo leaf capsule Dosage: 1 to 2 capsules/day, 3 times a day (Evidence level: very low, Recommendation level: Slightly recommended), Erigeron breviscapus tablets Dosage: 2 tablets/day, 3 times a day (evidence level: moderate. Recommendation level: strongly recommended), used for blood stasis and fluid retention syndrome.^[21,22,26,27,30]^

Supporting evidence [Ginkgo leaf tablets (capsule)]: RCTs have been conducted by Weng et al^[21]^ and Yang and Du^[22]^ where operations for glaucoma were taken in a controlled group compared with adding Ginkgo leaf capsule in experimental subjects group. The result showed better improvements in MD and eyesight. Evidence level: very low. (see Meta-analysis results--comparison 4 and SOF table-4). Ginkgo leaf capsule is traditional medicine for blood stasis and fluid retention syndrome. It is also low cost and national health insurance listed. It is easy to find and no adverse effect. Recommendation level: Slightly recommended.

Supporting evidence (Erigeron breviscapus tablets): Meta-analysis of 3 studies where fleabane was taken as experimental subjects compared with placebo in controlled group.^[26,27,30]^ The result showed better improvement in MS and vision in former group. Evidence level: moderate. (see Meta-analysis results--comparison 5 and SOF table-5). It is also low cost and national health insurance listed. It is easy to find and no adverse effect. Recommendation level: strongly recommended.

Wuling capsule Dosage: 3 capsules/time, twice a day (Level of evidence: Expert consensus, Intensity of recommendation: Weakly recommended); Shenling Baishu pills Dosage: 6 g/time, 3 times a day (Expert consensus), used for phlegm dampness syndrome.

Lycii and Chrysanthemi and Rehmanniae Bolus (water-honeyed pill) Dosage: 6 g/time, twice a day (Evidence level: very low, Recommendation level: Slightly recommended); Mingmu Dihuang Wan (water-honeyed pill) Dosage: 6 g/time, twice a day (Evidence level: very low, Recommendation level: Slightly recommended)^[31]^; Fuming tablets Dosage: 5 tabletes/time, three times a day (Evidence level: low, Recommendation level: Slightly recommended, used for syndrome of Yin deficiency of liver and kidney.^[28,38]^

Supporting evidence (Mingmu Dihuang Wan): RCTs have been conducted by Gao2013 where travoprost eye drops were taken in controlled group compared with experimental subjects travoprost eye drops and Mingmu Dihuang Wan.^[31]^ The result showed better improvements in MD and MS in latter group. Evidence level: very low. (see Meta-analysis results--comparison 6 and SOF table-6). Being traditional medicine for Yin deficiency of liver and kidney, Mingmu Dihuang Wan is low cost and easy to find without adverse effect. Recommendation level: Slightly recommended.

Supporting evidence (Fuming tablets): RCTs have been conducted by Li et al^[28]^ where Fuming tablets were taken as experimental subjects compared with mecobalamine in controlled group. The result showed better improvement in RNFL in experimental subjects. Evidence level: low. (see Meta-analysis results--comparison 7 and SOF table-7).

RCTs have been conducted by Wei and Wang^[38]^ where in controlled group, vitamin B1, mecobalamine, and intraocular depressurization were taken compared with additional Fuming tablets as experimental subjects. The result showed better improvement in the latter group in MD and intraocular pressure. Evidence level: very low. (see Meta-analysis results--comparison 8 and SOF table-8)

Being traditional medicine for Yin deficiency of liver and kidney, Fuming tablet is of low cost and easy to find without any adverse effect. Recommendation level: Slightly recommended.

### 3.3 Acupuncture therapy (expert consensus)

If the intraocular pressure was controlled within the target intraocular pressure value, acupuncture treatment can be assisted, especially for the patients with obvious visual function damage. Acupuncture treatment contributes to maintain and improve vision as well as expand the field of vision.

Main points: Jingming, Chengqi point, fish waist acupuncture point, Fengchi acupoints; Matching acupoints: Temple, Baihui, Sibai, Hoku;

Main acupoints: Shangjing Ming, Retrobulbar acupoint, Tongzi Liao, Wangu; Matching acupoints: Temple, Waiguan, Ganyu, Shenyu;

Main acupoints: Xiajing Ming, Sibai, Sizhu Kong, Tianzhu; Matching acupoints: Temple, Binao, Zusan Li, Sanyin Jiao.

The above groups applied alternately, or according to dialectical selection of acupuncture points, once a day.

### 3.4 Other results

Partial meta-analysis or qualitative analysis described the results of the study as positive, but the drugs used in the study were not recommended. See annex 6 for details.

## 4 Discussion

### 4.1 Main target users of the guideline

The onset of open angle glaucoma is usually occult, not easy to detect, and partial visual field defect has already occurred before a clear diagnosis. With the progression of the disease, there is further decline in vision and gradual narrowness in field of vision, which brings a great deal of inconvenience to the patient's daily life and adds immeasurable pain. More importantly, the optic nerve damage caused by glaucoma is irreversible, which makes high alert, early detection and positive control of further optic nerve damage very important in clinic. Chinese medicine may have some advantages in controlling the progression of visual field damage. Therefore, literatures about TCM treatment of open angle glaucoma were reviewed, on the basis of comprehensive retrieval of literatures, to be evaluated by a rigorous literature evaluation system. Chinese medicine ophthalmologists had discussed, on the basis of literature evaluation, the TCM dialectics and medication of this disease before the clinical validation and development of this guide, which provides clinicians guidance and reference to dialectical medication to make full use of Chinese medicine, alleviate patients’ sufferings, and save medical resources.

### 4.2 Study quality and limitations

The guideline mainly included RCTs, and literatures of non-RCTs were not yet included in the guideline because of their low level, such as case–control study, clinical experience reports, and so on; Patterns of syndrome listed in the guideline are common clinical syndrome types, not including all clinical syndromes. There commendations are rational, as they are based on both high-level studies and Expert consensus; however, more rigorous trials are still warranted for the identification of treatment effectiveness for POAG in the future.

### 4.3 Different treatments for the same disease in TCM

As TCM focuses on the whole body and patterns instead of diseases, patients with the same disease could be diagnosed as with different TCM patterns, in addition, due to the differences of academic philosophies, geographical location and patients’ physical constitution, and the fact that the formulae may vary for treating the same TCM pattern, although the treatment principles are the same. This guideline found that the TCM patterns of POAG were syndrome of stagnation of liver Qi, blood stasis and fluid retention syndrome, phlegm dampness syndrome, syndrome of Yin deficiency of liver and kidney; we first included clinical studies according to the evidence quality, and then conducted Expert consensus based on the clinical studies, thus sometimes recommending different formula for the same TCM pattern. We strongly recommend that the final clinical decision for each patient should be based on the combination of physician's experiences and patients’ individual situation.

## Acknowledgments

We thank the guideline writing group (ordered by the number of strokes of Chinese character of the family names), Guang Yang, Minglian Zhang, Lixia Zhang, Lixin Qiu, Chuanhong Jie, Lie Wu, Jian Zhou, Yue’e Tian, Jiajun Xu, Yanjiang Fu, Fengrong Li, Qiang Li, Chaoting Ma, Dandan Zhang, Nan Zhang, Yue Yang, Na Li, Yupeng He, Yan Wu.

We thank the ophthalmic experts with consensus who have contributed to the draft of this guideline (ordered by the number of strokes of Chinese character of the family names), Hongsheng Bi, Xiangdong Chen, Hui Deng, Junguo Duan, Yanjiang Fu, Jiansheng Gao, Ming Jin, Chuanhong Jie, Jianli Jiang, Zefeng Kang, Bo Li, Chuanke Li, Fengrong Li, Jing Liu, Qinghua Peng, Lixin Qiu, Zhankun Sun, Yue’e Tian, Tao Wang, Qiping Wei, Lie Wu, Xingwei Wu, Danlei Wu, Jiajun Xu, Jingsheng Yu, Jiachao Yan, Guang Yang, Jun Zhang, Minglian Zhang, Lixia Zhang, Jian Zhang, Xianghui Zhang, Yu Zhang, Jian Zhou, Zengyuan Zhuang, Mingkui Zeng, Ziming Zeng.

## Author contributions

**Conceptualization:** Y. Yang, Y. Yang.

**Data curation:** Q. Ma, W. Chen, Y. Yang, Y. Yang.

**Formal analysis:** Q. Ma, Y. Yang.

**Investigation:** C. Ma, W. Chen, Y. Yang.

**Methodology:** C. Ma, Q. Li, Y. He.

**Resources:** Q. Li, Q. Ma, W. Chen, Y. He.

**Supervision:** M. Jin.

**Validation:** M. Jin, Y. Yang.

**Visualization:** Q. Li.

**Writing – review & editing:** M. Jin, Y. Yang.

## Supplementary Material

Supplemental Digital Content
