# Supplementary material for: Evidence-based practice guideline of Chinese herbal medicine for primary open-angle glaucoma (qingfeng -neizhang)
Source: Medicine (Baltimore). 2018 Mar 30;97(13):e0126. doi: 10.1097/MD.0000000000010126 (PMC5895393; doi:10.1097/MD.0000000000010126)
Supplement: Supplemental Digital Content [file medi-97-e0126-s001.docx]

**Annex 1：Literature search expression**

Chinese literatures, CNKI as example, the expression of open angle glaucoma retrieval pattern is as follows;

# 1 Subject = Primary Open Angle Glaucoma or Subject = Open Angle Glaucoma or Subject = Simple Glaucoma or Subject = Bluish Glaucoma or Subject = Blue Blindness or Subject = Glaucoma (Exact match)

# 2 Subject = Chinese Medicine or Subject = Traditional Chinese Medicine or Subject = Prescription or Subject = Herbal Medicine or Subject = Chinese and Western Medicine or Subject = Proprietary Chinese Medicine or Subject = Traditional Medicine or Subject = Integrative Medicine or Subject = Alternative Therapy or Subject = Complementary Alternative Medicine or Subject = Acupuncture or Subject = Acupuncture (Exact match)

# 3 Full Text = Random Exclusion

(Title = Animal or Title = Mouse or Title = Dog or Title = Rabbit) (Exact match)

# 4 # 1 and # 2 and # 3

(2) English literatures, PubMed as an example, the expression of open angle glaucoma retrieval pattern is as follows;

#1 (Glaucoma,Open-Angle[mh]) OR (Glaucoma, Compensated) OR (Compensated Glaucoma) OR (Compensated Glaucomas) OR (Glaucomas, Compensated) OR (Glaucoma, Compensative) OR (Compensative Glaucoma) OR (Compensative Glaucomas) OR (Glaucomas, Compensative) OR (Glaucoma, Pigmentary) OR (Glaucomas, Pigmentary) OR (Pigmentary Glaucoma) OR (Pigmentary Glaucomas) OR (Glaucoma, Simple) OR (Glaucomas, Simple) OR (Simple Glaucoma) OR (Simple Glaucomas) OR (Open-Angle Glaucoma) OR (Glaucomas, Open-Angle) OR (Open-Angle Glaucomas) OR (Glaucoma Simplex)

#2 (Medicine, Chinese Traditional[mh]) OR (Traditional Chinese Medicine) OR (Chinese Medicine, Traditional) OR (Zhong Yi Xue) OR (Chinese Traditional Medicine) OR (Traditional Medicine, Chinese)

#3 acupuncture [mh]

#4 (acupuncture therapy [mh]) OR (therapy, acupuncture)

#5 #2 OR #3 OR #4

#６ #1 AND #5

#７ randomized controlled trial [pt]

#８ controlled clinical trial [pt]

#９ randomized [tiab]

#10 placebo [tiab]

#11 drug therapy [sh]

#12 randomly [tiab]

#13 trial [tiab]

#14 groups [tiab]

#15 #7 OR #8 OR #9 OR #10 OR #11 OR #12 OR #13 OR #14

#16 animals [mh] NOT humans [mh]

#17 #15 NOT #16

#15 #6 AND #17

| **Annex 2：Specialists Information** | | | | |
| --- | --- | --- | --- | --- |
| Name | Work Unit | Highest Education | Profression | Work Content |
| Yingxin Yang | Beijing Hospital of Traditional Chinese Medicine | PhD | Clinical Ophthalmologist | Framing the Overall Design and Organization of the Guideline |
| Qiuyan Ma | Beijing Hospital of Traditional Chinese Medicine | MD | Clinical Ophthalmologist | Document Retrieval, Evaluation, Composition of the Guideline |
| Yue Yang | Beijing Hospital of Traditional Chinese Medicine | MD | Clinical Ophthalmologist | Document Retrieval, Evaluation, Composition of the Guideline |
| Wei Chen | Evidence-Based Medicine Center at Beijing University of Traditional Chinese Medicine | PhD | Evidence-Based Medicine | Framing the Strategy in the Document Retrival, and Controlling the Methodological Quality |
| Hongsheng Bi | The Second Affiliated Hospital of Shandong University of Traditional Chinese Medicine | PhD | Clinical Ophthalmologist | Framing Recommendation Suggestion |
| Xiangdong Chen | The First Affiliated Hospital of Hunan University of Traditional Chinese Medicine | PhD | Clinical Ophthalmologist | Framing Recommendation Suggestion |
| Hui Deng | China-Japan Friendship Hospital | MD | Clinical Ophthalmologist | Framing Recommendation Suggestion |
| Junguo Duan | The Affiliated Hospital of Chengdu University of Traditional Chinese Medicine | PhD | Clinical Ophthalmologist | Framing Recommendation Suggestion |
| Yanjiang Fu | Beijing Hospital of Traditional Chinese Medicine | M.D. | Clinical Ophthalmologist | Framing Recommendation Suggestion |
| Jiansheng Gao | Eye Hospital China Academy of Chinese Medical Sciences | MD | Clinical Ophthalmologist | Framing Recommendation Suggestion |
| Ming Jin | China-Japan Friendship Hospital | MD | Clinical Ophthalmologist | Framing Recommendation Suggestion |
| Chuanhong Jie | Eye Hospital China Academy of Chinese Medical Sciences | PhD | Clinical Ophthalmologist | Framing Recommendation Suggestion |
| Jianli Jiang | The First Affiliated Hospital of Hunan University of Traditional Chinese Medicine | MD | Clinical Ophthalmologist | Framing Recommendation Suggestion |
| Zefeng Kang | Eye Hospital China Academy of Chinese Medical Sciences | PhD | Clinical Ophthalmologist | Framing Recommendation Suggestion |
| Bo Li | The First Affiliated Hospital of Hunan University of Traditional Chinese Medicine | MD | Clinical Ophthalmologist | Framing Recommendation Suggestion |
| Chuanke Li | The First Affiliated Hospital of Hunan University of Traditional Chinese Medicine | MD | Clinical Ophthalmologist | Framing Recommendation Suggestion |
| Fengrong Li | Beijing Hospital of Traditional Chinese Medicine | PhD | Clinical Ophthalmologist | Framing Recommendation Suggestion |
| Qiang Li | Beijing Hospital of Traditional Chinese Medicine | MD | Clinical Ophthalmologist | Framing Recommendation Suggestion |
| Jing Liu | Wangjing Hospital of China Academy of Chinese Medical Sciences | MD | Clinical Ophthalmologist | Framing Recommendation Suggestion |
| Chaoting Ma | Beijing Hospital of Traditional Chinese Medicine | MD | Clinical Ophthalmologist | Framing Recommendation Suggestion |
| Qinghua Peng | Hunan University Of Chinese Medicine | PhD | Clinical Ophthalmologist | Framing Recommendation Suggestion |
| Lixin Qiu | Beijing Tongren Hospital | MD | Clinical Ophthalmologist | Framing Recommendation Suggestion |
| Zhankun Sun | Shunyi Hospital of Beijing Hospital of Traditional Chinese Medicine | MD | Clinical Ophthalmologist | Framing Recommendation Suggestion |
| Yue'e Tian | Beijing Hospital of Traditional Chinese Medicine | MD | Clinical Ophthalmologist | Framing Recommendation Suggestion |
| Qiping Wei | Dongfang Hospital of Beijing University of Chinese Medicine | MD | Clinical Ophthalmologist | Framing Recommendation Suggestion |
| Lie Wu | Guanganmen Hospital of China Academy of Traditional Chinese Medicine | MD | Clinical Ophthalmologist | Framing Recommendation Suggestion |
| Xingwei Wu | Shanghai General Hospital | MD | Clinical Ophthalmologist | Framing Recommendation Suggestion |
| Jiajun Xu | Dongzhimen Hospital of Beijing University of Chinese Medicine | MD | Clinical Ophthalmologist | Framing Recommendation Suggestion |
| Jingsheng Yu | The First Affiliated Hospital of Hunan University of Traditional Chinese Medicine | MD | Clinical Ophthalmologist | Framing Recommendation Suggestion |
| Guang Yang | The First Teaching Hospital of Tianjin University of Traditional Chinese Medicine | PhD | Clinical Ophthalmologist | Framing Recommendation Suggestion |
| Jun Zhang | The Southern District at Guanganmen Hospital of China Academy of Traditional Chinese Medicine | MD | Clinical Ophthalmologist | Framing Recommendation Suggestion |
| Minglian Zhang | Xingtai Eye Hospital | PhD | Clinical Ophthalmologist | Framing Recommendation Suggestion |
| Mingliang Zhang | The First Affiliated Hospital of Hunan University of Traditional Chinese Medicine | MD | Clinical Ophthalmologist | Framing Recommendation Suggestion |
| Lixia Zhang | Eye Hospital China Academy of Chinese Medical Sciences | MD | Clinical Ophthalmologist | Framing Recommendation Suggestion |
| Jian Zhang | The First Affiliated Hospital of Hunan University of Traditional Chinese Medicine | MD | Clinical Ophthalmologist | Framing Recommendation Suggestion |
| Xianghui Zhang | The First Affiliated Hospital of Hunan University of Traditional Chinese Medicine | MD | Clinical Ophthalmologist | Framing Recommendation Suggestion |
| Yu Zhang | Guanganmen Hospital of China Academy of Traditional Chinese Medicine | MD | Clinical Ophthalmologist | Framing Recommendation Suggestion |
| Jian Zhou | Dongfang Hospital of Beijing University of Chinese Medicine | PhD | Clinical Ophthalmologist | Framing Recommendation Suggestion |
| Zengyuan Zhuang | Eye Hospital China Academy of Chinese Medical Sciences | MD | Clinical Ophthalmologist | Framing Recommendation Suggestion |
| Mingkui Zeng | The First Affiliated Hospital of Hunan University of Traditional Chinese Medicine | MD | Clinical Ophthalmologist | Framing Recommendation Suggestion |
| Ziming Zeng | The First Affiliated Hospital of Hunan University of Traditional Chinese Medicine | MD | Clinical Ophthalmologist | Framing Recommendation Suggestion |
| Tao Wang | Beijing Tongren Hospital | PhD | Clinical Ophthalmologist | Framing Recommendation Suggestion |
| Danlei Wu | Guanganmen Hospital of China Academy of Traditional Chinese Medicine | MD | Clinical Ophthalmologist | Framing Recommendation Suggestion |
| Jiachao Yan | The First Affiliated Hospital of Hunan University of Traditional Chinese Medicine | MD | Clinical Ophthalmologist | Framing Recommendation Suggestion |

**Annex 3:**

| Table 1. Characteristics of included randomized control trials | | | | | | | | | | | | | |
| --- | --- | --- | --- | --- | --- | --- | --- | --- | --- | --- | --- | --- | --- |
| **Study ID** | **Treatment group** | | | **Control group** | | | **Diagnostic criteria** | **Treatment** | | | **Control** | **Outcome measures** | **Adverse events** |
|  | **No. (M/F)** | **Mean/Range (years)** | **Eyes of treatment group (N)** | **No. (M/F)** | **Mean/Range (years)** | **Eyes of control group (N)** |  | **Intervention** | **Name of CHM^1^ investigated** | **Treatment period (Week)** |  |  |  |
| Zhang 2012^[14]^ | 14/9 | 44 | 40 | 11/10 | 46 | 36 | The early criteria of primary glaucoma recommended by the National Glaucoma Academic Group in 1987 | CHM plus the same co-intervention as in the control group | Self-prescribed Chinese herbal formula | 12 | Timolol Maleate Eye Drops | Vision,Glaucomatous visual field, Intraocular pressure | Not reported |
| Yao 2003^[15]^ | 19/11 | 40 | 30 | 18/10 | 43 | 28 | The early criteria of primary glaucoma recommended by the National Glaucoma Academic Group in 1987 | CHM plus the same co-intervention as in the control group | Self-prescribed Chinese herbal formula | 16 | Timolol Maleate Eye Drops | Vision,Glaucomatous visual field, Intraocular pressure | Not reported |
| Peng 2003^[16]^ | 20/14 | 45 | 61 | 20/14 | 44 | 62 | The early criteria of primary glaucoma recommended by the National Glaucoma Academic Group in 1987 | CHM plus the same co-intervention as in the control group | Self-prescribed Chinese herbal formula | 16 | Timolol Maleate Eye Drops | Vision,Glaucomatous visual field, Intraocular pressure | Not reported |
| Peng 1997^[17]^ | 21/31 | 55 | 78 | 16/23 | 54 | 54 | The diagnostic criteria of glaucoma in the book The Diagnosis and Treatment of Glaucoma | Post-surgery of glaucoma plus Qing-guang-an granules | Self-prescribed Chinese herbal formula | 7.2 | Post-surgery of glaucoma | Vision,Glaucomatous visual field, Intraocular pressure | Not reported |
| Luo 2012^[18]^ | not mentioned | 56.7 | 53 | not mentioned | 57 | 36 | not mentioned | Post-surgery of glaucoma+CHM | Self-prescribed Chinese herbal formula | 4 | Post-surgery of glaucoma | Vision,Glaucomatous visual field, Intraocular pressure | Not reported |
| Peng 1995^[19]^ | 25/6 | 19-36 | 62 | 14/3 | 19-33 | 34 | not mentioned | CHM | Self-prescribed Chinese herbal formula | 8 | Eye drops for lowering of intraocular pressure + Vitamin B1、ATP oral | Vision,Glaucomatous visual field, Intraocular pressure | Not reported |
| Peng 2004^[20]^ | 45/62 | 55 | 152 | 44/59 | 54 | 148 | The diagnostic criteria of glaucoma in the book The Diagnosis and Treatment of Glaucoma | CHM | Self-prescribed Chinese herbal formula | 6 | Placebo | Vision,Glaucomatous visual field | Not reported |
| Weng 2006^[21]^ | 8/10 | 26～72 | 30 | 7/12 | 30～70 | 30 | The early criteria of primary glaucoma recommended by the National Glaucoma Academic Group in 1987 | Post-surgery of glaucoma+vitamins+Ginkgo leaf capsule | Ginkgo leaf capsule 1 tablet Tid | 12 | Post-surgery of glaucoma+vitamins | Vision,Glaucomatous visual field, Intraocular pressure、P-VEP | No adverse event was found |
| Yang 2011^[22]^ | Not mentioned | 49 | 30 | Not mentioned | 49 | 30 | not mentioned | Post-surgery of glaucoma+ extract of Ginkgo Biloba Leaves | extract of Ginkgo Biloba Leaves injection 20ml ivgtt Qd×5d，+ Ginkgo leaf pills 5pills Tid | 24 | Post-surgery of glaucoma | Vision,Glaucomatous visual field, Intraocular pressure | No adverse event was found |
| Deng 2012^[23]^ | Not mentioned | 57.7 | 131 | Not mentioned | 58.4 | 133 | The early criteria of primary glaucoma recommended by the National Glaucoma Academic Group in 1987 | Ginkgo leaf capsule | Ginkgo leaf capsule 2pills Tid | 24 | Placebo 2pills Tid | Vision，visionary electrophysiology | Not reported |
| Shao 2008^[24]^ | 11/9 | 18～70 | 30 | 10/10 | 18～70 | 31 | not mentioned | Compound Xueshuantong capsule | Compound Xueshuantong capsule 3pills Tid | 12 | Vitamin C 200mg Tid | Vision,Glaucomatous visual field, Intraocular pressure | Not reported |
| Feng 2014^[25]^ | 12/14 | 56.7 | 40 | 11/13 | 57.1 | 35 | not mentioned | CHM plus the same co-intervention as in the control group | Yi-mai-kang pills(Erigeron breviscapus) 2pills Tid | 12 | Vitamin B12 | Mean visual acuity、Mean vision damage | Not reported |
| Ye 2003^[26]^ | 15/8 | 46.9 | 23 | 10/12 | 46.3 | 22 | not mentioned | CHM | Erigeron breviscapus 2pills Tid | 24 | Placebo 2 pills Tid | Corrected vision、Intraocular pressure、discus opticus (C/D ^2^)、automatic static vision threshold | Not reported |
| Wang 2004^[27]^ | 23/32 | 52.1 | 66 | 29/15 | 56.7 | 47 | not mentioned | CHM | Erigeron breviscapus 2 pills Tid | 24 | Placebo 2 pills Tid | Vision, Intraocular pressure | No adverse event was found |
| Li 2007^[28]^ | Not mentioned | 48.6 | 36 | Not mentioned | 48.6 | 29 | The early criteria of primary glaucoma recommended by the National Glaucoma Group in 1987 | CHM | Fuming tablets 5 tabletes Tid | 12 | Mecobalamin Tablets 1pill Tid | mfERG ^4^ and RNFL ^3^ | Not reported |
| Huang 2015^[29]^ | Not mentioned | 27～74 | 131 | Not mentioned | 27～74 | 131 | The early criteria of primary glaucoma recommended by the National Glaucoma Academic Group in 1987 | Liver and kidney yin deficiency syndrome to Lycii and Chrysanthemi and Rehmanniae Bolus plus the same co-intervention as in the control group | Lycii and Chrysanthemi and Rehmanniae Bolus | Not mentioned | Timolol Maleate Eye Drops | Vision,Glaucomatous visual field, Intraocular pressure | Not reported |
| Liu 2004^[30]^ | 12/6 | 47 | 20 | 9/9 | 55.3 | 19 | The early criteria of primary glaucoma recommended by the National Glaucoma Academic Group in 1987 | CHM | Erigeron breviscapus 2pills Tid | 24 | Placebo 2pills triple times per day | ,Glaucomatous visual field, Intraocular pressure, Mean retina sensitiveness | Not reported |
| Gao 2013^[31]^ | 26/18 | 46.3 | 44 | 23/21 | 48.5 | 44 | The early criteria of primary glaucoma recommended by the National Glaucoma Academic Group in 1987 | Liver kidney yin deficiency syndrome treated with Mingmu Dihuang Wan plus the same co-intervention as in the control group | Mingmu Dihuang Wan | 3 | Travoprost Eye Drops | Vision,Glaucomatous visual field, Intraocular pressure | Not reported |
| Jia 1994^[32]^ | 19/6 | 56.3 | 29 | 16/10 | 51 | 29 | not mentioned | Post-surgery of glaucoma+Qing-guang-kang pills | Self-prescribed Chinese herbal formula | 24 weeks | Post-surgery of glaucoma + placebo | vision, Intraocular pressure, eyesight, C/D | Not reported |
| Liu 1999^[33]^ | Not mentioned | 49.1 | 19 | Not mentioned | 55.8 | 15 | The early criteria of primary glaucoma recommended by the National Glaucoma Academic Group in 1987 | Ligustrazine capsule | Ligustrazine capsule 3pills Bid | 12 weeks | Placebo 3 pills triple times per day | Glaucomatous visual field, Intraocular pressure、Mean Damage | Not reported |
| Zhang 2006^[34]^ | 17/13 | 52.4 | 60 | 14/16 | 54.2 | 60 | not mentioned | Ligustrazine Injection | Ligustrazine Injection ivgtt Qd | Treatment group 6weeks；control group 8 weeks | Nimodipine 20mg Tid | Vision,Glaucomatous visual field, Intraocular pressure | Not reported |
| Yu 2009^[35]^ | not mentioned | 44.6 | 28 | not mentioned | 45 | 24 | not mentioned | CHM plus the same co-intervention as in the control group | Self-prescribed Chinese herbal formula | 5 weeks | Vitamin B1+Vitamin B12 | Vision,Glaucomatous visual field | Not reported |
| Liu 2012^[36]^ | 12/18 | 50.3 | 60 | 14/16 | 50.3 | 60 | The early criteria of primary glaucoma recommended by the National Glaucoma Academic Group in 1987 | Medlar, chrysanthemum and rehmannia granules | Self-prescribed Chinese herbal formula | 12 | Cobamamide Tablets 0.5g Tid | Vision,Glaucomatous visual field | Not reported |
| Zhang 2008^[37]^ | 15/13 | 54.4 | 48 | 15/13 | 53 | 47 | not mentioned | CHM plus the same co-intervention as in the control group | Self-prescribed Chinese herbal formula | 6 to 8 | Energy compound and nutrition- neurotherapy | Vision,Glaucomatous visual field | Not reported |
| Wei 2010^[38]^ | Not mentioned | 61.2 | 24 | Not mentioned | 61.2 | 28 Diagnostic criteria in Chinese Ophthalmology | The criteria from The Ophthalmology of China generated by the National Glaucoma Academic Group (Edited by Li. Fengming, et al.) | Group 1 Fuming tablets plus the same co-intervention as in the control group | Fuming tablets 5 tabletes Tid | 24 | Group 1 Vitamin B1 10mg Tid + Mecobalamin Capsules 0.5mg Tid | Vision,Glaucomatous visual field, Intraocular pressure | Not reported |
| Wang 2003^[39]^ | 29/31 | 49 | 60 | 31/29 | 48 | 60 | not mentioned | Puerarin eye drops | Puerarin eye drops | 3 | Timolol Maleate Eye Drops | Vision,Glaucomatous visual field, Intraocular pressure , conjunctival eyeground C/D | pain or sensation of foreign body. All symptoms were recovered automatically and the treatment had not been interrupted. |
| Dong 2013^[40]^ | 12/18 | 54.7 | 55 | 12/18 | 60.7 | 52 | not mentioned | CHM plus the same co-intervention as in the control group | Self-prescribed Chinese herbal formula | 12 | Eye drops for lowering of intraocular pressure | Vision,Glaucomatous visual field, Intraocular pressure, Mean sensitiveness, Mean Damage | Not reported |
| Xie 2012^[41]^ | 14/16 | 48 | 56 | 13/13 | 47 | 50 | The early criteria of primary glaucoma recommended by the National Glaucoma Academic Group in 1987 | Danzhixiaoyao drink plus the same co-intervention as in the control group | Danzhixiaoyao drink | 16 | Timolol Maleate Eye Drops | Vision,Glaucomatous visual field, Intraocular pressure 、Eye and body examinations | Not reported |
| Yang 2012^[42]^ | not mentioned | 56 | 88 | not mentioned | 55 | 80 | The criteria from The Book of Ophthalmology | CHM plus the same co-intervention as in the control group | Self-prescribed Chinese herbal formula | 12 | Travoprost Eye Drops | Vision,Glaucomatous visual field, vision Mean deviation | Not reported |
| Deng 2013^[43]^ | not mentioned | 55 | 30 | not mentioned | 56 | 30/20 | The criteria from Scholars' Comments: Diagnosis and Treatment of Primary Glaucoma | Group1 CHM plus the same co-intervention as in the control group | Self-prescribed Chinese herbal formula | 8 | Group1 Travoprost Eye Drops | Vision,Glaucomatous visual field, Intraocular pressure | Not reported |
| Xiao 2008^[44]^ | 52/46 | 50.6 | 98 | 48/44 | 50 | 92 | The early criteria of primary glaucoma recommended by the National Glaucoma Academic Group in 1987 | CHM plus the same co-intervention as in the control group | Self-prescribed Chinese herbal formula | 24 | Eye drops for lowering of intraocular pressure | Vision,Glaucomatous visual field, Intraocular pressure , optic nerves fiber thickness | Not reported |
| Li 2014^[45]^ | / | 48.2 | 44 |  | 48.2 | 44 | not mentioned | CHM | Self-prescribed Chinese herbal formula | 6 | Travoprost 1 tablet Tid | Glaucomatous visual field, vision | Not reported |
| Chen 2015^[46]^ | 18/22 | 33.4 | 40 | 21/19 | 30 | 40 | not mentioned | Bujing and yishi pills plus the same co-intervention as in the control group | Self-prescribed Chinese herbal formula | 24 | Travoprost 1 tablet Tid | Mean Damage, Mean Sensitiveness,visual field defect | Not reported |
| Li 2014^[47]^ | not mentioned | 60.9 | 51 | not mentioned | 61 | 47 | The criteria from The Ophthalmology of China generated by the National Glaucoma Academic Group (Edited by Li. Fengming, et al.) | Bujing and yishi pills plus the same co-intervention as in the control group | Self-prescribed Chinese herbal formula | 24 | Travoprost 1 tablet Tid | Mean Damage、Mean Sentitiveness | Not reported |
| Wu 2015^[48]^ | 12/10 | 21-63 | 40 | 10/9 | 20-65 | 32 | not mentioned | CHM | Self-prescribed Chinese herbal formula | 8~12 | Nutrition-neurotherapy +（Inoslne Tablets, Vitamin B 1-2 tablets tid；Travoprost 1tablet Tid） | Vision,Glaucomatous visual field, Intraocular pressure | Not reported |
| Li 2015^[49]^ | 21/22 | 63.4 | 43 | 22/21 | 63 | 43 | not mentioned | CHM plus the same co-intervention as in the control group | Self-prescribed Chinese herbal formula | 12 | Nutrition-neurotherapy （Vitamin B1, Vitamin B12, Inoslne Tablets） | Intraocular pressure 、Mean sensitivity, Mean Damage | Not reported |

CHM=Chinese herbal medicine, C/D=cup/disc area ratio, RNFL=retinal nerve fiber layer, ERG=electroretinogram.

Table 2.Compostions and details table of Chinese herbal medicine

| Name of CHM | Compostions | **Study ID** |
| --- | --- | --- |
| Self-prescribed Chinese herbal formula | Astragalus, Atractylodes, Angelica, Chuanxiong, Salvia, red peony medicine, Plantago, adzuki bean, Uncaria, Pueraria, Prunella. | Zhang 2012^[14]^ |
| Chinese medicine perscription for Glaucoma | Bupleurum, turmeric, Salvia, Zeeland, Achyranthes, Chuanxiong, Poria, Plantago, Alisma, chrysanthemum, white peony root, dodder and so on. | Yao 2003^[15]^ |
| Self-prescribed Chinese herbal formula | Bupleurum, turmeric, Salvia, Achyranthes, Chuan Shao, Citrus aurantium, Poria, Plantago, chrysanthemum, white peony root, dodder and so on. | Peng 2003^[16]^ |
| Qing-guang-an granules | Green Astragalus, habitat, Poria, Plantago, earthworm, red peony, safflower and other components. | Peng 1997^[17]^ |
| Self-prescribed Chinese herbal formula | Ligustrum lucidum, Wei Zi, Schisandra, Plantago, Dodder, Chuanxiong, Salvia, Angelica, Astragalus. | Luo 2012^[18]^ |
| Self-prescribed Chinese herbal formula | Earthworm, safflower, red peony root, Poria, Motherwort, Plantago. | Peng 1995^[19]^ |
| Qing-guang-an granules | Earthworm, red peony root, safflower, Poria, Plantago, Atractylodes, Astragalus, habitat made. | Peng 2004^[20]^ |
| Compound eliminating thrombosis capsule | Panax, Salvia, Astragalus, Scrophulariaceae | Shao 2008^[24]^ |
| Yi-mai-kang pills | Erigeron breviscapus | Feng 2014^[25]^、 |
| Lycii and Chrysanthemi and Rehmanniae Bolus | Rehmannia, dogwood (prepared），yam, tree peony bark, Poria, Alisma, medlar, chrysanthemum. | Huang 2015^[27]^ |
| Fuming pills | Chrysanthemum, cassia, Dendrobium, ginseng, medlar, dogwood (system), Rehmannia, berberine, antelope horn, stone Cassia, Prunella, wood and so on. | Li 2007^[28]^、Wei 2010^[38]^ |
| Mingmu Rehmannia Pill | Rehmannia, cornus meat, peony bark, yam, Poria, Alisma, medlar, chrysanthemum, Angelica, white peony root, Tribulus terrestris, Jieshi Cassia. Excipients for excipients honey. | Gao 2013^[31]^ |
| Qing-guang-kang pills | whole plant erigeron | Jia 1994^[32]^ |
| Tongqiao and mingmu IV | Bupleurum, Pueraria, Salvia, safflower, Angelica, windbreak, turmeric, medlar. | Yu 2009^[35]^ |
| Medlar, chrysanthemum and rehmannia granules | Medlar particles, chrysanthemum particles, Rehmannia particles, cornus particles, Epimedium particles, Alisma particles, Poria particles, Pueraria particles, Astragalus particles, Dan skin particles. | Liu 2012^[36]^ |
| Self-prescribed Chinese herbal formula | Health and Rehmannia, Angelica, white peony root, Chuan Shao, peach kernel, safflower, red peony root, earthworm, stone Cassia. | Zhang 2008^[37]^ |
| Salvia and notoginseng pills | Salvia miltiorriza, Panax notoginseng | Wei 2010^[38]^ |
| Self-prescribed Chinese herbal formula | Prunella, Pueraria, areca nut, Plantago, medlar, Polygonatum; Addition and subtraction: Chinese medicine plus square root, Salvia, Cyperus rotundus, mulberry leaves. | Dong 2013^[40]^ |
| Sterngthened Xiao-yao-yin soup | Angelica body, Atractylodes, Poria cocos, raw licorice shoots, white peony root, Bupleurum, fried gardenia, moutan. | Xie 2012^[41]^ |
| Sterngthened Xiao-yao-yin granules | Bupleurum, Poria, Atractylodes, Angelica, white peony root, licorice, chrysanthemum, medlar, moutan, gardenia, mint, Shichangpu. | Yang 2012^[42]^ |
| Self-prescribed Chinese herbal formula | Tribulus terrestris, Pueraria, Chuanxiong, turmeric, Ginkgo biloba, Shichangpu. | Xiao 2008^[44]^ |
| Chinese medicine Fuming soup | Shichangpu, Bupleurum, Moutan, Gardenia, Angelica. | Li 2014^[45]^ |
| Bujing and yishi pills | Green Tangerine peel, Salvia, Plantago, notoginseng, Chinese wolfberry, Su Wei Zi and so on | Chen 2015^[46]^、Li 2014^[47]^ |
| Self-prescribed Chinese herbal formula | Radix Astragali, Poria, Radix puerariae, Radix Ginseng, Habitat, Ligustrum lucidum, Radix, Salvia, Atractylodes, Turmeric, earthworm, Acorus gramineus, Chuanxiong, peach kernel. | Wu 2015^[48]^ |
| Self-prescribed Chinese herbal formula | Prunus persica or Prunus davidiana seed, Shichangpu, Chuanxiong, earthworm, turmeric, Salvia, Ophiopogon, Atractylodes, habitat, Yuan Senate, Ligustrum, Pueraria, Poria, Astragalus. | Li 2015^[49]^ |

**Annex 4: Meta analysis results**

**Comparsion 1 Self-prescribed Chinese herbal formula +Timolol eye drops +VitaminB1 VS Timolol eye drops +VitaminB1**

Outcome 1：intraocular pressure

Outcome 2：eyesight

Outcome 3：Visual field improvement rate

**Comparsion 2 Lycii and Chrysanthemi and Rehmanniae Bolus VS cobamamide**

Outcome 1：MD

Outcome 2：MS

Outcome 3：Visual field improvement rate

**Comparsion 3 Timolol eye drops + Lycii and Chrysanthemi and Rehmanniae Bolus compared to Timolol eye drops**

Outcome 1：MD

Outcome 2：MS

Outcome 3：intraocular pressure

**Comparsion 4 Operations for glaucoma +Ginkgo leaf capsule compared to Operations for glaucoma**

Outcome 1：eyesight

Outcome 2：MD

**Comparsion 5 Fleabane VS placebo**

Outcome 1:MS

Outcome 2: Visual field improvement rate

Outcome 3: intraocular pressure

Outcome 4:MD

**Comparsion 6 travoprost+** **Mingmu Rehmannia Pill compared to travoprost**

Outcome 1：MD

Outcome 2：MS

Outcome 3：intraocular pressure

**Comparsion 7 Fuming pills + mecobalamine VS mecobalamine**

Outcome 1：RNFL

**Comparsion 8 Fuming pills +VitaminB1+ mecobalamine compared to VitaminB1+ mecobalamine**

Outcome 1:MD

Outcome 2:MS

Outcome 3:intraocular pressure

**Annex 5: Quality assessment of evidence**

**SOF-Table1**

| **Self-prescribed Chinese herbal formula +VitaminB1 VS Timolol eye drops +VitaminB1 for primary open angle glaucoma** | | | | | | |
| --- | --- | --- | --- | --- | --- | --- |
| **Patient or population:** patients with primary open angle glaucoma **Settings:**  **Intervention:** Self-prescribed Chinese herbal formula +Timolol eye drops VS Timolol eye drops | | | | | | |
| **Outcomes** | **Illustrative comparative risks* (95% CI)** | | **Relative effect (95% CI)** | **No of Participants (studies)** | **Quality of the evidence (GRADE)** | **Comments** |
|  | Assumed risk | Corresponding risk |  |  |  |  |
|  | **Control** | **Self-prescribed Chinese herbal formula +Timolol eye drops +VitaminB1 VS Timolol eye drops +VitaminB1** |  |  |  |  |
| **intraocular pressure** ophthalmotonometer Follow-up: mean 16 weeks |  | The mean intraocular pressure in the intervention groups was **5.5 standard deviations lower** (6.34 to 4.65 lower) |  | 106 (1 study) | ⊕⊝⊝⊝ **very low**^1,2^ | SMD -5.5 (-6.34 to -4.65) |
| **eyesight** Manual test Follow-up: mean 16 weeks | **Study population** | | **RR 2.57**  (1.73 to 3.83) | 106 (1 study) | ⊕⊝⊝⊝ **very low**^2,3^ |  |
|  | **340 per 1000** | **874 per 1000** (588 to 1000) |  |  |  |  |
|  | **Medium risk population** | |  |  |  |  |
|  | **340 per 1000** | **874 per 1000** (588 to 1000) |  |  |  |  |
| **Visual field improvement rate** Campimeter+Manual test Follow-up: mean 16 weeks | **Study population** | | **RR 54.58**  (3.42 to 869.94) | 106 (1 study) | ⊕⊝⊝⊝ **very low**^2,4^ |  |
|  | **0 per 1000** | **0 per 1000** (0 to 0) |  |  |  |  |
|  | **Medium risk population** | |  |  |  |  |
|  | **0 per 1000** | **0 per 1000** (0 to 0) |  |  |  |  |
| *The basis for the **assumed risk** (e.g. the median control group risk across studies) is provided in footnotes. The **corresponding risk** (and its 95% confidence interval) is based on the assumed risk in the comparison group and the **relative effect** of the intervention (and its 95% CI). **CI=**Confidence interval, **RR=**Risk ratio. | | | | | | |
| GRADE Working Group grades of evidence **High quality:** Further research is very unlikely to change our confidence in the estimate of effect.  **Moderate quality:** Further research is likely to have an important impact on our confidence in the estimate of effect and may change the estimate. **Low quality:** Further research is very likely to have an important impact on our confidence in the estimate of effect and is likely to change the estimate. **Very low quality:** We are very uncertain about the estimate. | | | | | | |
| ^1^ Random trail not clear，Trail not clear，blinded-experiments were not taken，intraocular pressure is objective，but can be subjective too, high risk.  ^2^ Sample too few。 ^3^ Random trail not clear，Trail not clear，blinded-experiments were not taken， eyesight were tested manually，can be subjective，high risk。 ^4^ Random trail not clear，Trail not clear，blinded-experiments were not taken， Visual field improvement rate has some manual test，can be subjective，high risk。 | | | | | | |

**SOF-TABLE2**

| **Lycii and Chrysanthemi and Rehmanniae Bolus VS cobamamide for primary open angle glaucoma** | | | | | | |
| --- | --- | --- | --- | --- | --- | --- |
| **Patient or population:** patients with primary open angle glaucoma **Settings:**  **Intervention:** Lycii and Chrysanthemi and Rehmanniae Bolus VS cobamamide | | | | | | |
| **Outcomes** | **Illustrative comparative risks* (95% CI)** | | **Relative effect (95% CI)** | **No of Participants (studies)** | **Quality of the evidence (GRADE)** | **Comments** |
|  | Assumed risk | Corresponding risk |  |  |  |  |
|  | **Control** | **Lycii and Chrysanthemi and Rehmanniae Bolus VS cobamamide** |  |  |  |  |
| **MD** campimeter Follow-up: mean 12 weeks |  | The mean MD in the intervention groups was **0.13 lower** (1.2 lower to 0.94 higher) |  | 120 (1 study) | ⊕⊝⊝⊝ **very low**^1,2^ |  |
| **MS** campimeter Follow-up: mean 12 weeks |  | The mean MS in the intervention groups was **0 higher** (1.22 lower to 1.22 higher) |  | 120 (1 study) | ⊕⊝⊝⊝ **very low**^1,2^ |  |
| **Visual field improvement rate** campimeter+Manual test Follow-up: mean 12 weeks | **Study population** | | **RR 1.04**  (0.68 to 1.6) | 120 (1 study) | ⊕⊝⊝⊝ **very low**^1,2^ |  |
|  | **400 per 1000** | **416 per 1000** (272 to 640) |  |  |  |  |
|  | **Medium risk population** | |  |  |  |  |
|  | **400 per 1000** | **416 per 1000** (272 to 640) |  |  |  |  |
| *The basis for the **assumed risk** (e.g. the median control group risk across studies) is provided in footnotes. The **corresponding risk** (and its 95% confidence interval) is based on the assumed risk in the comparison group and the **relative effect** of the intervention (and its 95% CI). CI=Confidence interval, RR=Risk ratio. | | | | | | |
| GRADE Working Group grades of evidence **High quality:** Further research is very unlikely to change our confidence in the estimate of effect.  **Moderate quality:** Further research is likely to have an important impact on our confidence in the estimate of effect and may change the estimate. **Low quality:** Further research is very likely to have an important impact on our confidence in the estimate of effect and is likely to change the estimate. **Very low quality:** We are very uncertain about the estimate. | | | | | | |
| ^1^ Trail not clear， experimental objects and controlled group used different dosage forms，high risk in blinded-experiments，follow-up bias not clear. ^2^ Sample too few | | | | | | |

**SOF-TABLE3**

| **Timolol eye drops +** Lycii and Chrysanthemi and Rehmanniae Bolus **compared to Timolol eye drops for primary open angle glaucoma** | | | | | | |
| --- | --- | --- | --- | --- | --- | --- |
| **Patient or population:** patients with primary open angle glaucoma **Settings:**  **Intervention:** Timolol eye drops + Lycii and Chrysanthemi and Rehmanniae Bolus  **Comparison:** Timolol eye drops | | | | | | |
| **Outcomes** | **Illustrative comparative risks* (95% CI)** | | **Relative effect (95% CI)** | **No of Participants (studies)** | **Quality of the evidence (GRADE)** | **Comments** |
|  | Assumed risk | Corresponding risk |  |  |  |  |
|  | **Timolol eye drops** | **Timolol eye drops +** **Lycii and Chrysanthemi and Rehmanniae Bolus** |  |  |  |  |
| **MD** campimeter |  | The mean MD in the intervention groups was **1.09 lower** (1.5 to 0.68 lower) |  | 262 (1 study) | ⊕⊝⊝⊝ **very low**^1,2^ |  |
| **MS** campimeter |  | The mean MS in the intervention groups was **1.37 higher** (0.82 to 1.92 higher) |  | 262 (1 study) | ⊕⊝⊝⊝ **very low**^1,2^ |  |
| **intraocular pressure** ophthalmotonometer |  | The mean intraocular pressure in the intervention groups was **4.01 lower** (4.81 to 3.21 lower) |  | 262 (1 study) | ⊕⊝⊝⊝ **very low**^1,2^ |  |
| *The basis for the **assumed risk** (e.g. the median control group risk across studies) is provided in footnotes. The **corresponding risk** (and its 95% confidence interval) is based on the assumed risk in the comparison group and the **relative effect** of the intervention (and its 95% CI). CI=Confidence interval. | | | | | | |
| GRADE Working Group grades of evidence **High quality:** Further research is very unlikely to change our confidence in the estimate of effect.  **Moderate quality:** Further research is likely to have an important impact on our confidence in the estimate of effect and may change the estimate. **Low quality:** Further research is very likely to have an important impact on our confidence in the estimate of effect and is likely to change the estimate. **Very low quality:** We are very uncertain about the estimate. | | | | | | |
| ^1^ Random trail not clear，Trail not clear，high risk in blinded-experiments，follow-up bias not clear。 ^2^ Sample too few。 | | | | | | |

**SOF-TABLE4**

| **Operations for glaucoma +Ginkgo leaf capsule compared to Operations for glaucoma** | | | | | | |
| --- | --- | --- | --- | --- | --- | --- |
| **Patient or population:** patients with primary open angle glaucoma **Settings:**  **Intervention:** Operations for glaucoma +Ginkgo leaf capsule  **Comparison:** Operations for glaucoma | | | | | | |
| **Outcomes** | **Illustrative comparative risks* (95% CI)** | | **Relative effect (95% CI)** | **No of Participants (studies)** | **Quality of the evidence (GRADE)** | **Comments** |
|  | Assumed risk | Corresponding risk |  |  |  |  |
|  | **Operations for glaucoma** | **Operations for glaucoma +Ginkgo leaf capsule** |  |  |  |  |
| **eyesight** Manual test Follow-up: 12-24 weeks | **Study population** | | **RR 2.88**  (1.41 to 5.87) | 120 (2 studies) | ⊕⊝⊝⊝ **very low**^1,2^ |  |
|  | **133 per 1000** | **383 per 1000** (188 to 781) |  |  |  |  |
|  | **Medium risk population** | |  |  |  |  |
|  | **133 per 1000** | **383 per 1000** (188 to 781) |  |  |  |  |
| **MD** campimeter Follow-up: 12-24 weeks |  | The mean MD in the intervention groups was **10.38 lower** (16.13 to 4.63 lower) |  | 120 (2 studies) | ⊕⊝⊝⊝ **very low**^1,2^ |  |
| *The basis for the **assumed risk** (e.g. the median control group risk across studies) is provided in footnotes. The **corresponding risk** (and its 95% confidence interval) is based on the assumed risk in the comparison group and the **relative effect** of the intervention (and its 95% CI). CI=Confidence interval, RR=Risk ratio. | | | | | | |
| GRADE Working Group grades of evidence **High quality:** Further research is very unlikely to change our confidence in the estimate of effect.  **Moderate quality:** Further research is likely to have an important impact on our confidence in the estimate of effect and may change the estimate. **Low quality:** Further research is very likely to have an important impact on our confidence in the estimate of effect and is likely to change the estimate. **Very low quality:** We are very uncertain about the estimate. | | | | | | |
| ^1^ Random trail not clear，Trail not clear，blinded-experiments were not taken，high risk in blinded-experiments，follow up not clear. ^2^ Sample too few | | | | | | |

**SOF-TABLE5**

| **Erigeron breviscapus tablets VS placebo for primary open angle glaucoma** | | | | | | |
| --- | --- | --- | --- | --- | --- | --- |
| **Patient or population:** patients with primary open angle glaucoma **Settings:**  **Intervention:** Erigeron breviscapus tablets VS placebo | | | | | | |
| **Outcomes** | **Illustrative comparative risks* (95% CI)** | | **Relative effect (95% CI)** | **No of Participants (studies)** | **Quality of the evidence (GRADE)** | **Comments** |
|  | Assumed risk | Corresponding risk |  |  |  |  |
|  | **Control** | **Erigeron breviscapus tablets VS placebo** |  |  |  |  |
| **MS** campimeter Follow-up: mean 24 weeks | **Study population** | | **RR 2.89**  (0.77 to 10.76) | 186 (3 studies) | ⊕⊕⊕⊝ **moderate**^1^ |  |
|  | **104 per 1000** | **301 per 1000** (80 to 1000) |  |  |  |  |
|  | **Medium risk population** | |  |  |  |  |
|  | **91 per 1000** | **263 per 1000** (70 to 979) |  |  |  |  |
| **Visual field improvement rate** campimeter Follow-up: mean 24 weeks | **Study population** | | **RR 10.47**  (3 to 36.51) | 197 (3 studies) | ⊕⊕⊕⊝ **moderate**^1^ |  |
|  | **23 per 1000** | **241 per 1000** (69 to 840) |  |  |  |  |
|  | **Medium risk population** | |  |  |  |  |
|  | **46 per 1000** | **482 per 1000** (138 to 1000) |  |  |  |  |
| **intraocular pressure** ophthalmotonometer Follow-up: mean 24 weeks |  | The mean intraocular pressure in the intervention groups was **0.71 higher** (1.54 lower to 2.96 higher) |  | 158 (2 studies) | ⊕⊕⊝⊝ **low**^1,2^ |  |
| **MD** campimeter Follow-up: mean 24 weeks |  | The mean MD in the intervention groups was **2.4 higher** (0.22 to 4.58 higher) |  | 113 (1 study) | ⊕⊕⊝⊝ **low**^1,2^ |  |
| *The basis for the **assumed risk** (e.g. the median control group risk across studies) is provided in footnotes. The **corresponding risk** (and its 95% confidence interval) is based on the assumed risk in the comparison group and the **relative effect** of the intervention (and its 95% CI). CI=Confidence interval, RR=Risk ratio. | | | | | | |
| GRADE Working Group grades of evidence **High quality:** Further research is very unlikely to change our confidence in the estimate of effect.  **Moderate quality:** Further research is likely to have an important impact on our confidence in the estimate of effect and may change the estimate. **Low quality:** Further research is very likely to have an important impact on our confidence in the estimate of effect and is likely to change the estimate. **Very low quality:** We are very uncertain about the estimate. | | | | | | |
| ^1^ partial studies, Random trail not clear，Trail not clear。 ^2^ Sample too few | | | | | | |

**SOF-TABLE6**

| **travoprost+Mingmu Dihuang Wan compared to travoprost for primary open angle glaucoma** | | | | | | |
| --- | --- | --- | --- | --- | --- | --- |
| **Patient or population:** patients with primary open angle glaucoma **Settings:**  **Intervention:** travoprost + Mingmu Dihuang Wan  **Comparison:** travoprost | | | | | | |
| **Outcomes** | **Illustrative comparative risks* (95% CI)** | | **Relative effect (95% CI)** | **No of Participants (studies)** | **Quality of the evidence (GRADE)** | **Comments** |
|  | Assumed risk | Corresponding risk |  |  |  |  |
|  | **travoprost** | **travoprost+ Mingmu Dihuang Wan** |  |  |  |  |
| **MD** campimeter Follow-up: mean 3 weeks |  | The mean MD in the intervention groups was **6.4 lower** (8.53 to 4.27 lower) |  | 88 (1 study) | ⊕⊝⊝⊝ **very low**^1,2^ |  |
| **MS** campimeter Follow-up: mean 3 weeks |  | The mean MS in the intervention groups was **9 higher** (7.53 to 10.47 higher) |  | 88 (1 study) | ⊕⊝⊝⊝ **very low**^1,2^ |  |
| **intraocular pressure** ophthalmotonometer Follow-up: mean 3 weeks |  | The mean intraocular pressure in the intervention groups was **1.1 lower** (2.52 lower to 0.32 higher) |  | 88 (1 study) | ⊕⊝⊝⊝ **very low**^2^ |  |
| *The basis for the **assumed risk** (e.g. the median control group risk across studies) is provided in footnotes. The **corresponding risk** (and its 95% confidence interval) is based on the assumed risk in the comparison group and the **relative effect** of the intervention (and its 95% CI). **CI=**Confidence interval; | | | | | | |
| GRADE Working Group grades of evidence **High quality:** Further research is very unlikely to change our confidence in the estimate of effect.  **Moderate quality:** Further research is likely to have an important impact on our confidence in the estimate of effect and may change the estimate. **Low quality:** Further research is very likely to have an important impact on our confidence in the estimate of effect and is likely to change the estimate. **Very low quality:** We are very uncertain about the estimate. | | | | | | |
| ^1^ Trail not clear，blinded-experiments were not taken， MD、MS、intraocular pressure can be subjective, high risk in blinded-experiments。 follow-up bias not clear。 ^2^ Sample too few。 | | | | | | |

**SOF-TABLE 7**

| **Fuming tablets + mecobalamine VS mecobalamine for primary open angle glaucoma** | | | | | | |
| --- | --- | --- | --- | --- | --- | --- |
| **Patient or population:** patients with primary open angle glaucoma **Settings:**  **Intervention:** Fuming tablets + mecobalamine VS mecobalamine | | | | | | |
| **Outcomes** | **Illustrative comparative risks* (95% CI)** | | **Relative effect (95% CI)** | **No of Participants (studies)** | **Quality of the evidence (GRADE)** | **Comments** |
|  | Assumed risk | Corresponding risk |  |  |  |  |
|  | **Control** | **Fuming tablets + mecobalamine VS mecobalamine** |  |  |  |  |
| **RNFL** Optical coherence tomography Follow-up: mean 12 weeks |  | The mean RNFL in the intervention groups was **0.07 higher** (0.01 to 0.13 higher) |  | 65 (1 study) | ⊕⊕⊝⊝ **low**^1,2^ |  |
| *The basis for the **assumed risk** (e.g. the median control group risk across studies) is provided in footnotes. The **corresponding risk** (and its 95% confidence interval) is based on the assumed risk in the comparison group and the **relative effect** of the intervention (and its 95% CI). **CI=**Confidence interval; | | | | | | |
| GRADE Working Group grades of evidence **High quality:** Further research is very unlikely to change our confidence in the estimate of effect.  **Moderate quality:** Further research is likely to have an important impact on our confidence in the estimate of effect and may change the estimate. **Low quality:** Further research is very likely to have an important impact on our confidence in the estimate of effect and is likely to change the estimate. **Very low quality:** We are very uncertain about the estimate. | | | | | | |
| ^1^ Random trail not clear，Trail not clear，blinded-experiments were not taken， RNFL is objective，low risk in blinded-experiments ^2^ Sample too few。 | | | | | | |

**SOF-TABLE8**

| **Fuming tablets +VitaminB1+ mecobalamine compared to VitaminB1+ mecobalamine for primary open angle glaucoma** | | | | | | |
| --- | --- | --- | --- | --- | --- | --- |
| **Patient or population:** patients with primary open angle glaucoma **Settings:**  **Intervention:** Fuming tablets +VitaminB1+ mecobalamine **Comparison:** VitaminB1+ mecobalamine | | | | | | |
| **Outcomes** | **Illustrative comparative risks* (95% CI)** | | **Relative effect (95% CI)** | **No of Participants (studies)** | **Quality of the evidence (GRADE)** | **Comments** |
|  | Assumed risk | Corresponding risk |  |  |  |  |
|  | **VitaminB1+ mecobalamine** | **Fuming** **tablets +VitaminB1+ mecobalamine** |  |  |  |  |
| **MD** campimeter Follow-up: mean 24 weeks |  | The mean MD in the intervention groups was **1.11 lower** (2.09 to 0.13 lower) |  | 52 (1 study) | ⊕⊝⊝⊝ **very low**^1,2^ |  |
| **MS** campimeter Follow-up: mean 24 weeks |  | The mean MS in the intervention groups was **0.66 higher** (0.5 lower to 1.82 higher) |  | 52 (1 study) | ⊕⊕⊝⊝ **low**^1,2^ |  |
| **intraocular pressure** ophthalmotonometer Follow-up: mean 24 weeks |  | The mean intraocular pressure in the intervention groups was **2.42 lower** (4.33 to 0.51 lower) |  | 52 (1 study) | ⊕⊝⊝⊝ **very low**^2^ |  |
| *The basis for the **assumed risk** (e.g. the median control group risk across studies) is provided in footnotes. The **corresponding risk** (and its 95% confidence interval) is based on the assumed risk in the comparison group and the **relative effect** of the intervention (and its 95% CI). **CI=**Confidence interval; | | | | | | |
| GRADE Working Group grades of evidence **High quality:** Further research is very unlikely to change our confidence in the estimate of effect.  **Moderate quality:** Further research is likely to have an important impact on our confidence in the estimate of effect and may change the estimate. **Low quality:** Further research is very likely to have an important impact on our confidence in the estimate of effect and is likely to change the estimate. **Very low quality:** We are very uncertain about the estimate. | | | | | | |
| ^1^ Random trail not clear，Trail not clear，blinded-experiments were not taken，MD、MS、intraocular pressure can be subjective, high risk in blinded-experiments。 ^2^ Sample too few。 | | | | | | |

| **Annex 6:**  **Table 1. Chinese herbal medicine not recommended for primary open angle glaucoma** | | | | | | | |  |
| --- | --- | --- | --- | --- | --- | --- | --- | --- |
| **Experimental Object** | **Experimental Objects VS Controlled Group** | **Target** | **Mean [Deviation] (MD) /Risk Ratio (RR) [95% Confidence Interval (CI)]** | **P value** | **Evidence Source** | **Evidence Level** | **Reason** | |
| Chinese herbal medicine for activating blood and reactivating blood | Chinese herbal medicine for activating blood and reactivating blood +intraocular depressurization eye drops **VS** intraocular depressurization eye drops | MD | MD -2.79[-4.54, -1.03] | *p*=0.002 | 4 studies^[14～16，44]^ of meta analysis | Very Low | New informal presciption. Low evidence level.Not supported by clinic experts. not recommended. | |
|  |  | MS | MD 8.6[7.57, 9.64] | *p*<0.00001 | 2 studies^[15，16]^ of meta analysis | Very Low |  |  |
|  |  | Intraocular Pressure | MD -1.27[-2.02, -0.52] | *p*=0.001 | 3 ^[14～16]^ studies of meta analysis | Very Low |  |  |
|  | Chinese herbal medicine for activating blood and reactivating blood +Operations for glaucoma **VS** Operations for glaucoma | Visual field improvement rate | RR 13.59 [5.47，33.77] | *p*<0.00001 | 2 studies^[17，18]^ of meta analysis | Very Low | New informal presciption. Low evidence level.Not supported by clinic experts. not recommended. | |
|  |  | Eyesight | RR 4.19 [2.80, 6.28] | *p*<0.00001 |  | Very Low |  |  |
|  | Chinese herbal medicine for activating blood and reactivating blood **VS** placebo | Visual field improvement rate | RR 8.88 [5.43，14.50] | *p*<0.00001 | 2 studies^[20，32]^ of meta analysis | Very Low | New informal presciption. Low evidence level.Not supported by clinic experts. not recommended. | |
|  |  | Eyesight | RR 2.51[2.06, 3.04] | *p*<0.00001 |  | Very Low |  |  |
|  | Chinese herbal medicine for activating blood and reactivating blood **VS** intraocular depressurization eye drops+ VitaminB1、ATP | Visual field improvement rate、 | RR 23.89 [1.49, 382.47] | *p*=0.02 | Peng1995^[15]^ | Very Low | New informal presciption. Low evidence level.Not supported by clinic experts. not recommended. | |
|  |  | Eyesight | RR(95%CI)4.66 [2.23, 9.72] | *p*<0.0001 |  | Very Low |  |  |
|  |  | Intraocular Pressure | MD -6.33 [-6.56, -6.10] | *p*<0.00001 |  | Very Low |  |  |
| Chinese medicine for tonifying kidney and activating blood | Chinese medicine for tonifying kidney and activating blood +trophic nerve **VS** trophic nerve | MD | MD -3.50 [-5.27, -1.73] | *p*=0.003 | 5 studies^[35，37，46～47，49]^ of meta analysis | Very Low | New informal presciption. Low evidence level.Not supported by clinic experts. not recommended. | |
|  |  | MS | MD 3.06[1.10, 5.02] | *p*=0.002 |  | Very Low |  |  |
|  | Tongqiao and mingmu IV+Vitamin B1+VitaminB12 **VS** VitaminB1+VitaminB12 | Eyesight | RR 1.71[1.17,2.51] | *p*=0.006 | Yu2009^[35]^ | Very Low | New informal presciption. Low evidence level.Not supported by clinic experts. not recommended. | |
| Promoting Qi flow and soothing liver chinese medicine | Self-prescribed Chinese herbal formula+intraocular depressurization eye drops **VS** intraocular depressurization eye drops | MD | MD -3.54 [-5.51, -1.57] | *p*=0.0004 | Dong2013^[40]^ | Very Low | New informal presciption. Low evidence level.Not supported by clinic experts. not recommended. | |
|  |  | MS | MD 4.18 [1.97, 6.39] | *p*=0.0002 |  | Very Low |  |  |
|  |  | Eyesight | MD 0.16 [0.04, 0.27] | *p*=0.007 |  | Very Low |  |  |
|  | Self-prescribed Chinese herbal formula **VS** mecobalamine | MD | MD -3.80 [-6.16, -1.44] | *p*=0.002 | Li2014^[45]^ | Very Low | New informal presciption. Low evidence level.Not supported by clinic experts. not recommended. | |
| Chinese medicine for invigorating qi, activating blood and absorbing clots | Self-prescribed Chinese herbal formula **VS** trophic nerve | MD | MD -2.70 [-4.69, -0.71] | *p*=0.008 | Wu2015^[48]^ | Very Low | New informal presciption. Low evidence level.Not supported by clinic experts. not recommended. | |
|  |  | Eyesight | RR 1.10 [0.50, 2.41] | *p*=0.81 |  | Very Low |  |  |
| Proprietary Chinese medicine | Compound Xueshuantong capsule **VS** VitaminC | MD | MD -3.50 [-5.27, -1.73] | *p*=0.0001 | Shao2008^[24]^ | Very Low | New informal presciption. Low evidence level.Not supported by clinic experts. not recommended. | |
|  |  | MS | MD 3.10 [1.29, 4.91] | *p*=0.0008 |  | Very Low |  |  |
|  | Ligustrazine Injection **VS** Nimodipine | MS | MD -2.65 [-3.05, -2.25] | *p*<0.00001 | Zhang2006^[34]^ | Very Low | New informal presciption. Low evidence level.Not supported by clinic experts. not recommended. | |

MD=Mean Deviation, RR=Risk Ratio, CI=95% Confidence Interval.
